# 3 Dimensional photonic scans for measuring body volume and muscle mass in the standing horse

**DOI:** 10.1371/journal.pone.0229656

**Published:** 2020-02-27

**Authors:** Stephanie J. Valberg, Amanda K. Borer Matsui, Anna M. Firshman, Lauren Bookbinder, Scott A. Katzman, Carrie J. Finno

**Affiliations:** 1 McPhail Equine Performance Center, Department of Large Animal Clinical Sciences, Michigan State University, East Lansing, MI, United States of America; 2 Department of Veterinary Population Medicine, College of Veterinary Medicine, University of Minnesota, St. Paul, MN, United States of America; 3 Department of Surgical and Radiological Sciences, University of California Davis, Davis, CA, United States of America; 4 Department of Population Health and Reproduction, University of California Davis, Davis, CA, United States of America; University College Dublin, School of Veterinary Medicine, IRELAND

## Abstract

**Reasons for performing study:**

Although muscle mass strongly influences performance, there is currently no effective means to measure the 3-dimensional muscle mass of horses. We evaluated a 3-dimensional (3D) scanning methodology for its ability to quantify torso and hindquarter volumes as a proxy for regional muscle mass in horses.

**Objectives:**

Determine the repeatability of 3D scanning volume (V) measurements and their correlation to body weight, estimated body volume and muscle/fat ultrasound (US) depth.

**Methods:**

Handheld 3D photonic scans were performed on 16 Quarter Horses of known body weight 56 days apart (n = 32 scans) with each scan performed in duplicate (n = 32 replicates). Tail head fat, gluteal and longissimus dorsi muscle depths were measured using US. Processed scans were cropped to isolate hindquarter (above hock, caudal to tuber coxae) and torso (hindquarter plus dorsal thoracolumbar region) segments and algorithms used to calculate V. Torso and hindquarter volume were correlated with body weight and US using Pearson’s correlation and with estimated torso volume (50% body weight / body density) with Bland-Altman analysis.

**Results:**

Scans took 2 min with < 3.5% error for duplicate scans. Torso volume (R = 0.90, P< 0.001) and hindquarter volume (R = 0.82, P< 0.001) strongly correlated with body weight and estimated BV (R = 0.91) with low bias. Torso volume moderately correlated to mean muscle US depth (R = 0.4, P< 0.05) and tail head fat (R = 0.42, P< 0.01). Mean muscle US depth moderately correlated to body weight (R = 0.50, P< 0.01).

**Main limitations:**

3D Scans determine body volume not muscle volume.

**Conclusions:**

The hand-held 3D scan provided a rapid repeatable assessment of torso and hindquarter volume strongly correlated to body weight and estimated volume. Superimposition of regional scans and volume measures could provide a practical means to follow muscle development when tail head fat depth remain constant.

## Introduction

The volume (V) of the locomotor muscles in terms of number of fibers and their architectural arrangements exert a profound influence on performance by impacting the power generated by muscle at varying velocities of shortening [1; 2]. In particular, the large proximal pelvic muscles generate much of the force required for equine athletic performance [1]. Athletic horses are reported to have greater muscle mass (53–57%) when compared to other horses (42%) and a larger portion of their overall muscle weight endowed in the propulsive locomotor muscles of the hindlimb region [3]. Development of specific muscle groups is colloquially known to be characteristic of specific equine performance types, however, there are few if any scientific reports documenting gross development of muscle groups critical for particular equine disciplines (Pub Med and Google Scholar search 01/09/2018)[1].

The paucity of information related to muscle mass and body V in horses is related to the technical difficulties in measuring these parameters [1; 2]. There has been no readily available mechanism to quantify 3D muscle mass in horses and to follow the changes that occur as horses progress through training. Conventionally, photographs, body condition scoring and ultrasonography (US) have been used to as a proxy to assess muscle mass in research studies of horses [2; 4; 5]. The subjectivity of body condition scoring and the limited number of muscles that can be assessed in 2 dimensions with US impede the accurate measurement of muscle development in the entire body [2; 5; 6]. Magnetic resonance imaging (MRI) is capable of providing a 3D assessment of muscle mass, however, it is has not yet been used to report body V or muscle mass in horses and is neither readily available, nor affordable for routine follow up [7].

The need for accurate measurements of human body shape and body dimensions for retail and commercial purposes has resulted in the development of digitized optical methods to generate 3D photonic images of an individual [8–11]. Commercial scans use numerous stationary lasers within a booth to provide 3D body contours of people within a 20 second period [8; 11]. Working toward our long term goal of devising a rapid means to accurately assess body V and muscle development in the horse, we adapted a handheld infrared photonic scanner to produce a 3D image of a horse [12]. The handheld Optical Structure Sensor Scanner projected a speckled pattern of invisible infrared light and captured distortions in the projection as a 3D mesh. Post scan processing algorithms were used to transform the mesh into a solid body where volumes could be measured.

We hypothesized that the 3D photonic scan would be highly repeatable and provide a good correlate to body V in horses. The purpose of the present study was to assess the repeatability of the developed 3D scanning technique and to determine how well volumetric measures reflected body weight as well as muscle and fat mass assessed using US. To more specifically assess muscle V important for propulsion, cropping of scans was performed to isolate regions such as the hindquarter above the hock and the torso (dorsal thoracolumbar region + hindquarter).

## Materials and methods

### Validation of volume assessment

In order to determine that the 3D scanner accurately assesses volume, two boxes of known volume (0.0571 m^3^ and 0.0213 m^3^) were stacked askew on top of each other to form a more complex structure and the volume of the combined box structure assessed using the 3 D scanner (S1 Fig). The scanning was repeated 4 times and the percent error calculated for each assessment.

### Horses

Sixteen unfit horses of Quarter Horse-related breeds, 10 mares, 6 geldings, with a mean age of 12.1 ± 2.7 years housed at a University facility on dry lots were used in the present study. Body condition scores (1–9) ranged from 4 to 7 with a mean (SD) of 5.6 ± 0.9. Body weights (BW) were obtained at the time of each body scan. To increase the number of technical scan replicates, the scanning process was repeated on the same 16 horses 56 days apart as part of an ongoing nutritional study. The research was approved by IACUC at the University of California, Davis and Michigan State University in compliance with the US National Research Council's Guide for the Care and Use of Laboratory Animals, the US Public Health Service's Policy on Humane Care and Use of Laboratory Animals, and Guide for the Care and Use of Laboratory Animals. The individuals assisting with the research in this manuscript have given written informed consent (as outlined in PLOS consent form) to publish their images.

## Scanning

### Procedure

**Fig 1:**
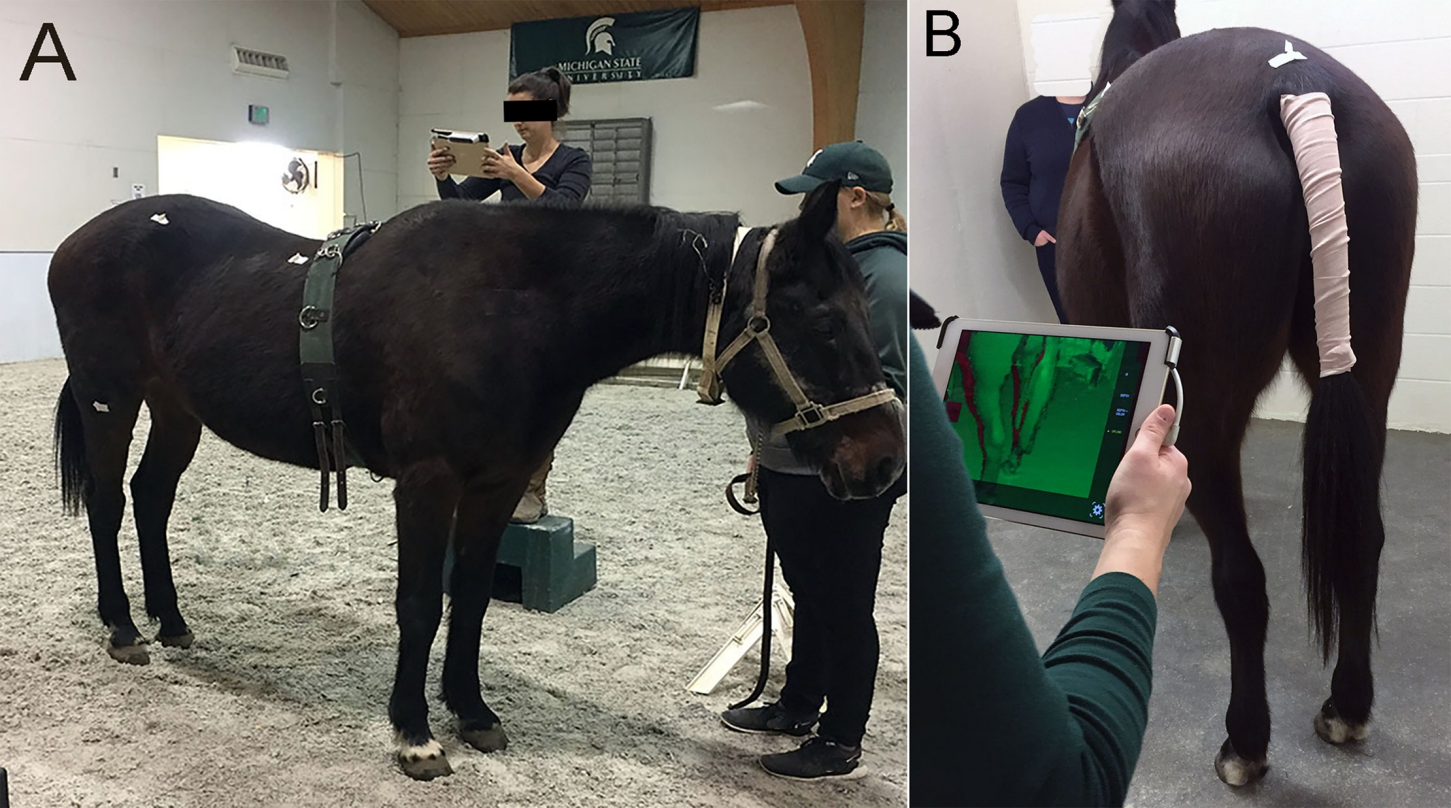
3D scanning process. A. Positioning of the horse for obtaining a 3D scan using an iPad with occipital structure sensor scanner and surcingle with anatomic markers. B. Diagonal position of the horse for scanning in a stall and screen view of the scanning image.

Horses were groomed, the tail was wrapped and 6 cm pieces of white tape partially folded in half were affixed to the skin at the highest point on the tuber coxae and over the dorsal spinous process of the most caudal sacral vertebra. A lunging surcingle was fitted into the natural girth groove to define the cranial margin of the torso (Fig 1A). Xylazine hydrochloride (0.3–0.4 mg/kg IV) was administered to any horses that were reluctant to stand still. Horses were positioned so that both forelimbs were square, both hindlimbs were square and all four limbs were placed naturally underneath the body fully weight bearing. Duplicate scans were performed within minutes of each other with horses standing in the same squared position. During positioning, horses were facing one corner diagonally in a 3.7m x 3.7 m stall (Fig 1B). Two mounting blocks 0.45 m in width were placed equidistant between the fore and hindlimbs approximately 1.5–2 m from the horse on the left and right sides (Fig 1A).

An Occipital Structure Sensor (ST01, Occipital, Inc., Boulder, CO) was attached to an iPad Air 2 (Model A1566, Apple, Cupertino CA) running the Structure application (Structure v1.9, Occipital Inc, Boulder CO)^2^ (Fig 1A). The iPad was then linked via a wireless router (Linksys E2500, Irvine CA)^3^ to a laptop (Dell Precision 7520, 7520, Intel^®^ Core^™^ i7-7920HQ CPU @ 3.10 Gz, 64 GB RAM, Dell, Round Rock, TX,) sitting outside the stall running the scanning program (Skanect Pro v1.9 (Win64, Occipital, San Francisco, CA). The scan was started with the operator holding the iPad with attached scanner slightly above chest height beginning at the left front of the horse and progressing caudally at a steady smooth pace (Fig 1B, S1 Video). The procedure included stepping up and down on the mounting block to scan the dorsal left torso (Fig 1A), moving from left to right sides behind the horse (Fig 1B), stepping up and down the mounting block on the right side and finishing at the right shoulder (S1 Video). Whenever possible while scanning, one of the two mounting blocks or set of hooves were kept in the scan to facilitate tracking (Fig 1B). Each scan took approximately 2 min. Scans were stopped, discarded and repeated if the scan lost tracking, if the horse moved during the scanning process or if the left and right halves of the surcingle did not align perfectly over the back in the scan. On every occasion that a scan was performed, a second scan was obtained to assess accuracy. The second scan was performed within 15 min of the first complete scan with the horse again placed in a squared stance with hindlimbs directly underneath the horse. Hindquarter and torso V for each horse were comprised of the mean of both scans.

### Post-processing of scans

**Fig 2:**
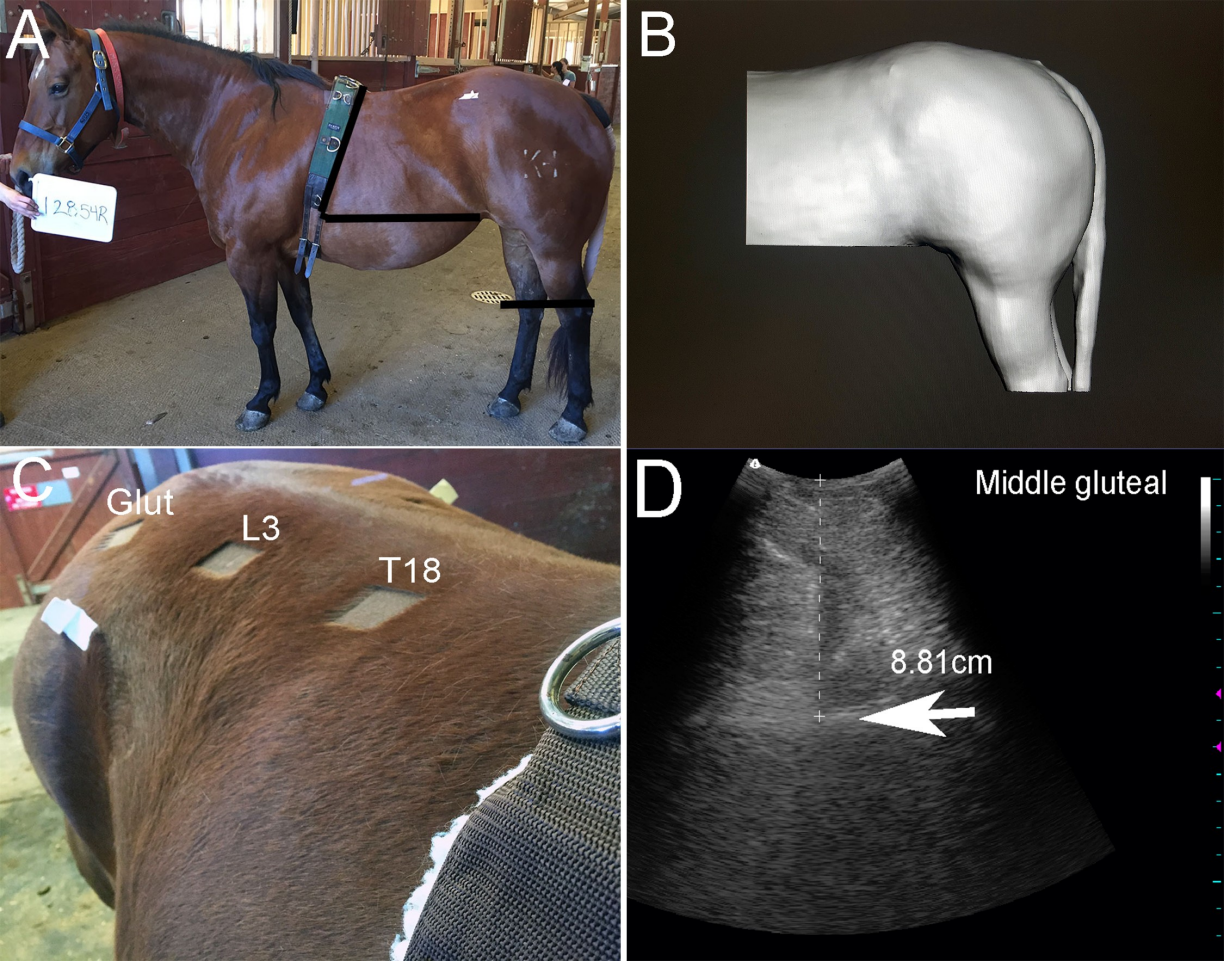
Torso and hindquarter volume cropping and processing of ultrasound images. A. Regions of the body that were cropped (black lines) in order to obtain the torso volume measurement. The torso was defined as the area caudal to the surcingle above a horizontal plane drawn parallel to the floor from the skin fold at the juncture of the stifle and flank and included the hindquarters above the point of the hock. B. The 3D image of the body scan of the torso of the horse in A. C. The 3 areas utilized for US imaging including T18, L3 over the longissimus muscle and middle gluteal muscle. D. US image of the middle gluteal muscle where muscle depth was measured for the superficial compartment. Arrow indicates fascia separating superficial and deep middle gluteal compartments.

Object files for each scan were exported from Skanect and imported into the Meshmixer program (Meshmixer, Version 3.3.15, Autodesk, Inc., San Francisco, CA). The horse’s body was then isolated in each scan by cropping the handler, walls, mounting block and ground off the mesh. The horse’s body was then cropped to isolate the ‘torso’. The torso was delineated cranially by the surcingle, and ventrally by a plane drawn parallel to the floor from the junction of the flank and stifle to the surcingle. Cropping of the lower abdomen minimized the volume in the region of the large colon. The torso included the HQ which was cropped above the point of the hock with the tail removed if necessary, to assess the full semimembranosus/tendinosus area (Fig 2A and 2B, S2 Video). The hindquarter sector was isolated by cropping the torso at markers placed on the highest point of the tuber coxae. The width of the mounting block in duplicate scans was evaluated as a control object.

## Ultrasonography

Horses were restrained in stocks and US was performed by one experienced ultrasonographer (AMF) using a TeraVet 3000 Ultrasound machine (Teratech Corp, Burlington MA). US was performed one the same day as scanning was performed. Standard skin preparation consisted of clipping the hair, cleaning with alcohol and application of US gel.

### T18 and L3 lumbar muscles

Left and right lumbar muscles at the level of the 18^th^ thoracic vertebrae were found by palpating the curvature of the 18^th^ rib craniodorsally to the point at which it could be palpated connecting to the spinal column. Left and right lumbar muscles (longissimus dorsi/ cranial gluteus medius) at the level of the 3^rd^ lumbar vertebra were found by following a line that ran directly vertical towards midline from the caudal most aspect of the 18^th^ rib. For both of these locations a 5cm square of hair was clipped at the level of T18 and L3, the center of which was 10 cm from the dorsal midline (in the iliocostal muscle groove) (Fig 2C).

A curvilinear probe (Terason 5C2A-Vet Convex 5.0–2.0 MHz) was oriented transversely following the skin curvature and 3 separate images were captured that depicted the skin surface, longissimus dorsi and margin of the rib or transverse process. Because little subcutaneous fat was evident, muscle depths were measured from skin surface to the bone margin.

### Middle gluteal

The middle gluteal muscle depth was measured on left and right sides at a location equidistant between the dorsal most aspect of the tuber sacrale and the dorsal most aspect of the tuber coxae (Fig 2D). A 5 cm square of hair was clipped at the midpoint of this line. The curvilinear probe was oriented transversely and muscle depth was measured in three separate images from the skin surface to the fascial plane that separates the gluteal medius’ superficial and deep (gluteus accessorius) compartments (Fig 2D).

### Fat pad

Subcutaneous adipose tissue was measured at a site 5 cm to the left and right of the root of the tail. A linear 6-MHz probe (Terason 12L5-Vet) was oriented transversely and fat depth was measured from the skin surface to the ventral limit of the subcutaneous adipose tissue.

### Statistical analysis

Scan V and US measurements were tested for normality using D'Agostino & Pearson omnibus normality test and found to be normally distributed. Mean and standard deviations of scan V, BW and US depths were calculated. Percent error was calculated for torso V and hindquarter V by dividing the difference in volume between scan 1 and scan 2 by scan 1 and multiplying by 100. The coefficient of variations for US measurements were calculated from the 3 measurements of US depth taken for each horse at each site. Pearson’s Correlation coefficients were calculated to assess relationships among BW, torso V, hindquarter V and US depths. Bland Altman plots were analyzed to compare 3D scan V for torso with estimated V for the torso. Estimated torso V was calculated as (BW/ (body density X 0.5)), based on the fact that torso V was approximately ½ of the horse’s body V in our preliminary scans of the entire horse. The value used for body density was approximated from previous lean human and horse references (1010 kg/m^3^) [2; 13; 14]. Statistical analyses were performed using GraphPad Prism 7.0 (Graphpad Software, La Jolla, CA).Results with P <0.05 were reported as statistically significant.

## Results

### Validation of volume assessment

The known volume of the stacked boxes was 0.07834 m^3^. The mean volume for 4 repeated 3D scans of the boxes was 0.07667 ± 0.0004 m^3^. The mean percent error was 2.1 ± 0.56%.

### Volume and percent error

**Fig 3:**
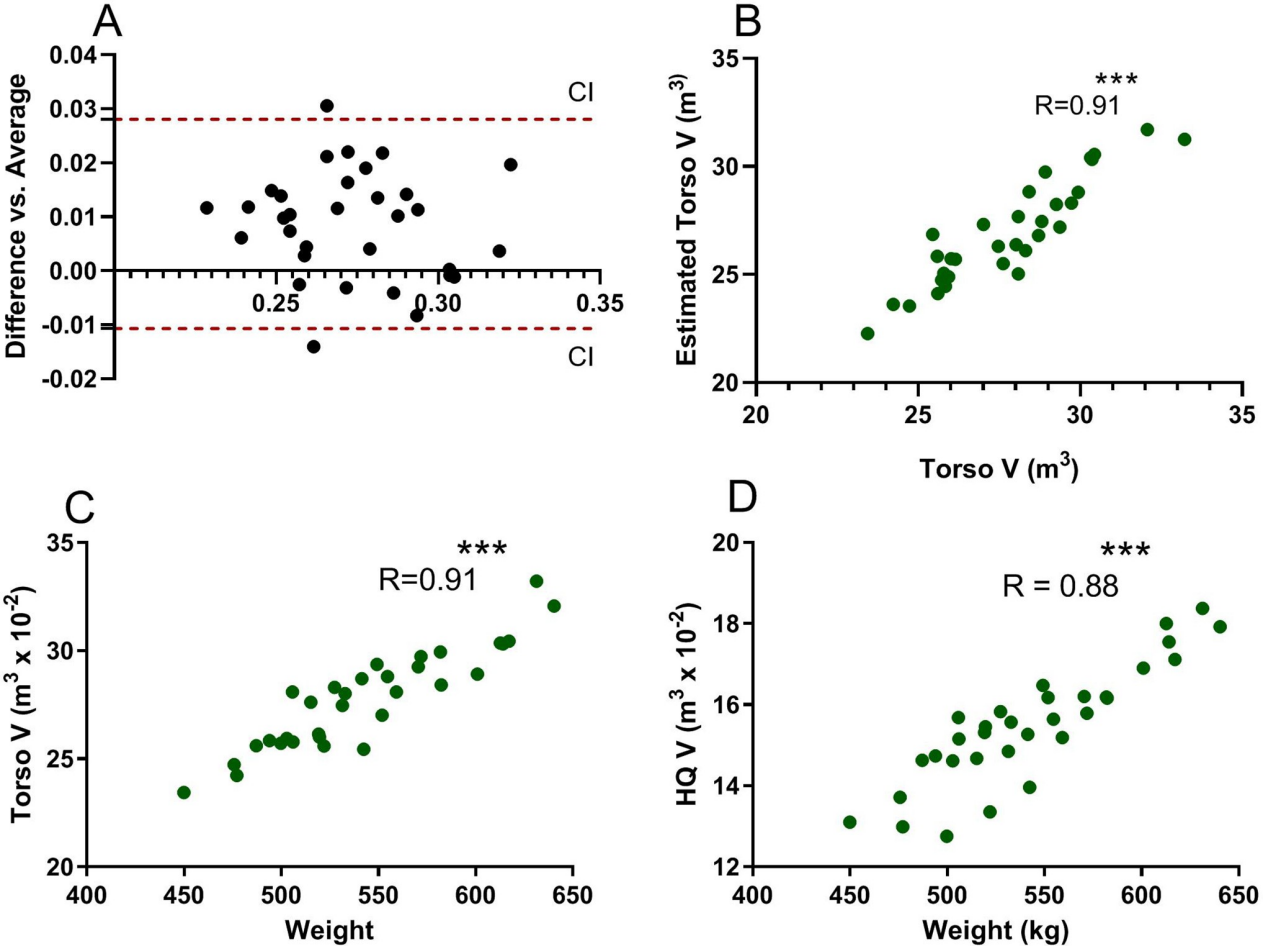
Accuracy of 3D scanning to assess body volume. A. Bland Altman plot comparing the differences between torso V and average of estimated torso volume (V) with 95% confidence intervals depicted by red dashed lines. Only two of 32 V measures were outside of the 95% confidence limits. B. Correlation of 3D scan torso V with estimated torso V (R = 0.91). C. Positive correlation of 3D scan torso volume with body weight. D. Positive correlation of 3D scan hindquarter volume with body weight. ***P<0.001.

Mean torso V was 0.2777 ± 0.0229 m^3^ and mean estimated torso V was 0.2690 ± 0.0241 m^3^. The mean difference between duplicates was low at 0.0075 ± 0.0099 m^3^ (CI 0.0051–0.0123) with little estimated bias indicated by Bland Altman analysis (Fig 3A). There was a high degree of correlation between torso V and estimated torso V (R = 0.91) (Fig 3B). The error between scans was low at 3.0 ± 2.1% for torso V and 3.5 ± 3.3% for hindquarter V. Duplicate measurements of the width of the control object in the scan had an error of 2.6 ± 1.9%.

### Correlation to body weight

**Fig 4:**
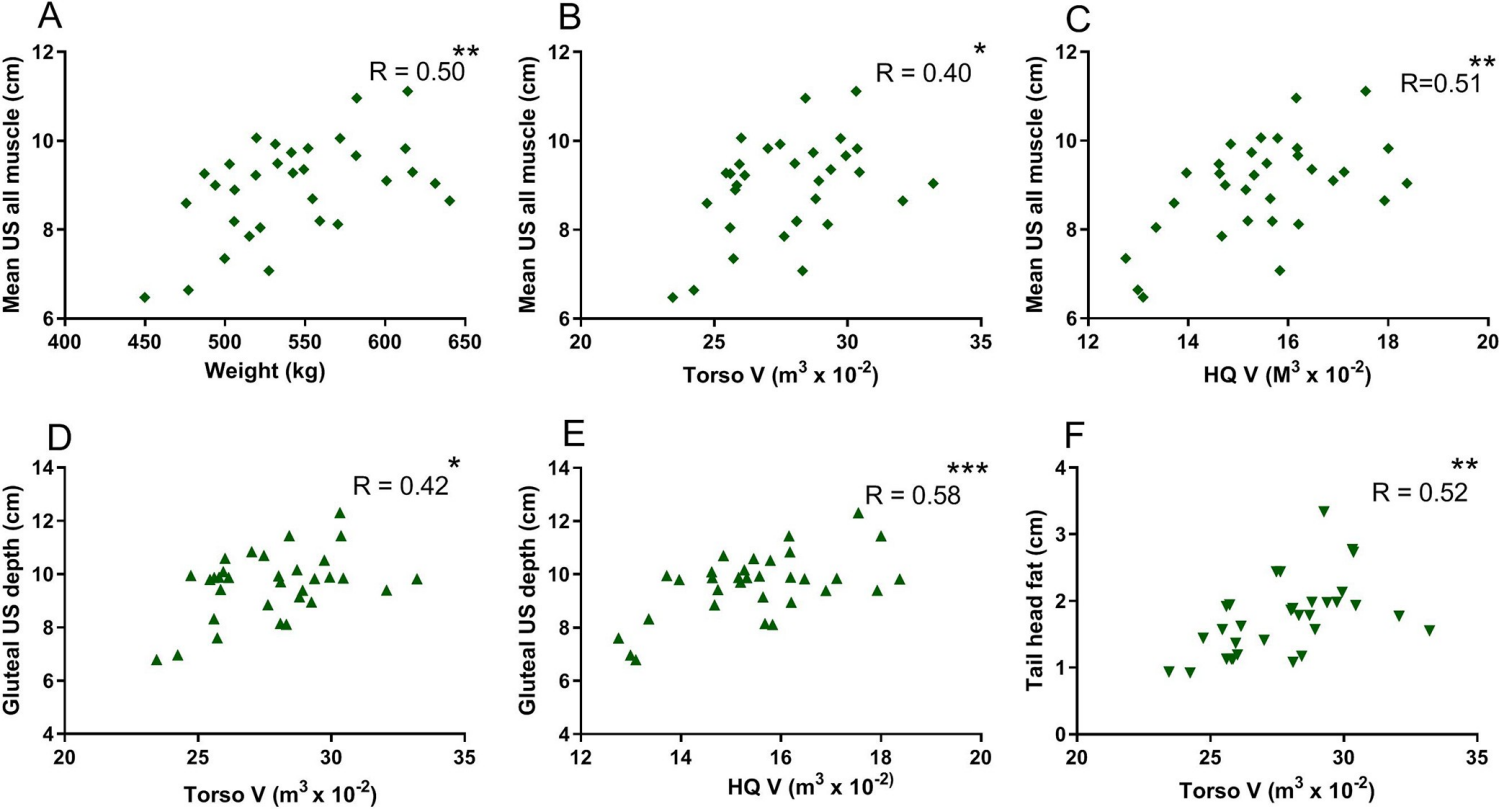
Correlation and Pearson’s correlation coefficients. A. Mean US measures of two sites in lumbar muscle and one site over the middle gluteal muscle compared to body weight. B. Mean US values for the three muscle depths compared to torso V. C. Mean US values for the three muscle depths compared to hindquarter (HQ) V. D. Positive correlation of middle gluteal ultrasound depth with torso V. E. Positive correlation of middle gluteal ultrasound depth with HQ V. F. Positive correlation of tail head fat to torso V. * P<0.05, ** P<0.01, ***P<0.001.

There was a strong positive correlation between torso V and BW (R = 0.91, P<0.0001) as well as hindquarter V and BW (R = 0.88, P<0.0001) (BW range 450 to 640 kg) (Fig 3C and 3D). The mean of T18, L3 and gluteal muscle US depth was moderately positively correlated to BW (R = 0.50, P = 0.004) (Fig 4A). Tail head fat depth was not correlated to body weight (R = 0.18, P = 0.3).

### Scanning volume versus ultrasound depth

The coefficient of variation for US depth at each site ranged from 1.73 to 3.45%, with the lowest variation found for L3 and gluteal muscle (Table 1). Torso V (R = 0.40, P = 0.02) and hindquarter V (R = 0.51, P = 0.002) showed a moderate positive correlation to the mean of T18, L3 and gluteal muscle US depth (Fig 4B and 4C). Gluteal muscle depth had stronger positive correlations to torso V and hindquarter V (Fig 4D and 4E) than T18 (R = 0.12, P = 0.1, hindquarter V, R = 0.30, P = 0.5 Torso V) or L3 muscle depths. Torso V, but not hindquarter V (R = 0.13, P = 0.5), was moderately correlated to tail head fat (Fig 4F).

**Table 1:** Mean (SD) ultrasound depths at the sites; T18 and L3 of the left (L) and right (R) longissimus muscles, the middle gluteal muscle and the tail head (fat).

	T18		L3		Gluteal		Tail head	
	L	R	L	R	L	R	L	R
**Depth (cm)**	**7.59 ± 1.13**	**7.90 ± 1.20**	**9.37 ± 1.53**	**9.63 ± 1.27**	**9.57 ± 1.33**	**9.67 ± 1.26**	**1.70 ± 0.56**	**1.79 ± 0.60**
**CV (%)**	**3.35 ± 2.10**	**3.24 ± 1.98**	**1.75 ± 1.02**	**1.87 ± 1.0**	**1.73 ± 1.01**	**2.18 ± 1.47**	**3.45 ± 2.29**	**2.83 ± 1.89**

The coefficient of variation (CV) expressed as a percent was calculated as the SD for 3 measurements at each site divided by the mean depth.

## Discussion

The present study determined that the hand-held 3D scanning methodology provided a rapid method to measure torso and hindquarter V in horses with a high degree of repeatability. The <3.5% error between duplicate scans was similar to errors reported for more expensive stationary laser 3D scanning used in human studies.[8; 11] Scans took approximately 2 min to perform with horses standing still. The initial challenge in performing scans was to ensure that the scan maintained tracking throughout. Utilizing a steady pace for movement of the handheld scanner around the horse, ensuring objects such as paired hooves or mounting blocks were always in the scan and using a surcingle to ensure that left and right sides perfectly aligned were all useful adaptations to ensure high quality scans.

Validation of a new method of measurement requires comparison to the gold standard. In the case of equine body V, however, there are no previous technologies that have accurately assessed body V in the horse to the best of our knowledge. In humans, the `gold standard' for assessing body volume is hydrostatic weighing, which is impractical for horses as it requires immersion in a water tank.[9; 10] Other methods to assess muscle and fat body composition in humans include dual energy X-ray absorption (DEXA), air displacement, bio-electrical impedance analysis and MRI, however, validated data for these techniques is not available for horses for comparison.[2; 10]

In order to determine if our 3D scan V measurements reasonably reflected actual body V, we first scanned a structure (boxes) of known volume and found the scan provided an accurate volume assessment with 2% error. Next, we compared measured torso V to a best estimate of body V calculated as BW divided by body density. Fat tissue (0.90) is less dense than bone, muscle tissue (1.1 to 1.3) and water (1.0).[15] We utilized an estimated body density from human studies of 1010 kg/m^3^ and preliminary data that showed our torso V represented approximately 50% of the entire horse’s body V (Fig 2).[13; 14] Very small mean differences between measured torso V and estimated BV were found with mean values differing by < 3.1%. A strong correlation was found between torso V and estimated torso V (R = 0.91). A Bland Altman analysis demonstrated that the limit of agreement between methods was narrow and without bias. In addition, there was a strong positive correlation (R = 0.91) between BW and both torso V and hindquarter V. Thus, to the best of our abilities, we were able to confirm that the handheld 3D infrared scan appears to provide an accurate measure of body V in horses.

As our long term goal is to use 3D Scanning to follow muscle development, we compared US assessment of muscle mass with BW and body V calculations. Mean values for US assessment of combined lumbar and middle gluteal muscles were moderately correlated to torso V (accounting for only 16% of variability) and hindquarter V (25% of variability). Mean US depth was not as strongly correlated to BW (accounting for 25% of the variability) as torso V (83% of variability) or hindquarter V (77% of variability). This was not an unexpected finding since US only provides two dimensions of muscle size at a limited site. The advantage of 3D scanning would appear to be the ability to rapidly capture the full dimension of muscle bulk compared to US. The advantage of US measurement, however, is that it direct measures skeletal muscle depth and excludes subcutaneous fat, bone mass, lung volume, gastrointestinal fill and potentially hydration status that are incorporated into measures of body V.[2; 5; 10]

In the present study, 3D scans were cropped in order to focus on the propulsive hindlimb muscles. The lower abdomen, limbs below the hock, forelimb, head and neck of the horse were removed from measurements. The stifle fold was used as a readily identifiable plane to crop the lower abdomen to minimize the impact of gastrointestinal volume. To further remove an impact of large intestinal volume, more of the abdomen could be cropped, however, this would require highly standardized affixed markers to avoid potential variability. Using the cropping methods in the present study, torso V and hindquarter V were significantly correlated to muscle US depth. Importantly however, the impact of subcutaneous fat on torso V was readily evident based on significant correlation of torso V to US tail head fat depth.

Fat occupies a disproportionate volume compare to muscle due to its lower density. At higher body condition scores, body fat deposition increases exponentially, equally distributed between internal and external sites.[2; 6] Both intermuscular and subcutaneous fat deposits are more strongly correlated to total fat deposits than intraabdominal fat.[6] Thus, comparisons of 3D scans for muscle development in an individual horse over time must include a measure of body fat to ensure that changes in V are not a result of increased fat deposition. We utilized the tail head region to assess fat depth because there was little observable subcutaneous fat deposited in the longissimus and gluteal muscles regions evaluated with US. This site had low coefficient of variation. Other studies have measured B mode fat depth at a site 5 cm lateral from the midline at the center of the pelvic bone with reported correlation coefficients between actual and ultrasound-measured rump fat thickness ranging from R^2^ = 0.90 to 0.96. [16; 17] The specific site of measurement of fat on the hindquarter should be clearly described as it is unclear from Westervelt what the center of the pelvic bone specifically represents.[16]

The results of the present study suggest that 3D scanning V could be of great benefit in assessing athletic horses and are supported by a recent study of 3D scanning used to assess rowing performance in humans.[18] Overall, studies of rowers found that absolute, rather than proportional measurements, and 2D and 3D rather than 1D measurements were the best predictors of rowing ergometry performance, with whole body V and surface area, standing height, mass and leg length being the strongest individual predictors. In addition, the study found that scanning was time-efficient and noninvasive, enhancing participation and providing a historical record of each athlete at a particular point in time that could be reexamined in the future without the athlete present.[18] All of these are features that would be of value in assessing equine athletes.

To be clear, the scanning technique used in the present study assesses volume and is not a direct measure of muscle mass. US depth can be used as a proxy for the mass of specific muscles.[5] US depth, however, was not strongly correlated to hindquarter V and US has the disadvantages of variability of measurements between different individuals, time required to US numerous muscles and the potential need to clip horses.[5] In contrast, a 2 min scan is highly repeatable, quick and encompasses the entire superficial muscle contour. One could argue that because body weight was strongly correlated to estimated body volume, body weight could be used rather than a body scan to estimate volume. An estimated body volume, however, would not provide a means to assess changes in the volume of specific body regions with training. In contrast, a scan could be further divided into sectors, such as left versus right hindquarters, which would provide more specific indications of regional muscle development. Thus, while US and body weight are currently useful proxies for assessing muscle development, 3 dimensional scanning has the potential to provide additional information on regional muscle development.

In conclusion, the handheld Occipital Structure Sensor Scanner and post-processing algorithms provided a rapid accurate means to assess body V in horses that was highly proportional to BW. Results suggest it would be feasible to utilize this technology to follow muscle development of an individual horse over time using cropped scans provided that control measures are taken to ensure changes in V do not reflect changes in fat deposition, hydration status, large intestinal fill, hydration or posture.

## Supporting information

S1 Fig Image from the scan of the two boxes used to validate the 3D scanner's ability to accurately assess volume. (TIF)

S1 File Previous utilization of the scanning technique to assess body volume in the horse. (PDF)

S1 Video The procedure for performing a 3D photonic scan. The process begins at the horse’s right shoulder and finishing at the horse’s left shoulder.
(MP4)

S2 Video Video depicting the 3D scanned image of the torso from many different angles after processing and cropping. (MP4)
