# Supplementary figures and images for: 3 Dimensional photonic scans for measuring body volume and muscle mass in the standing horse
Source: PLoS One. 2020 Feb 27;15(2):e0229656. doi: 10.1371/journal.pone.0229656 (PMC7046215; doi:10.1371/journal.pone.0229656)

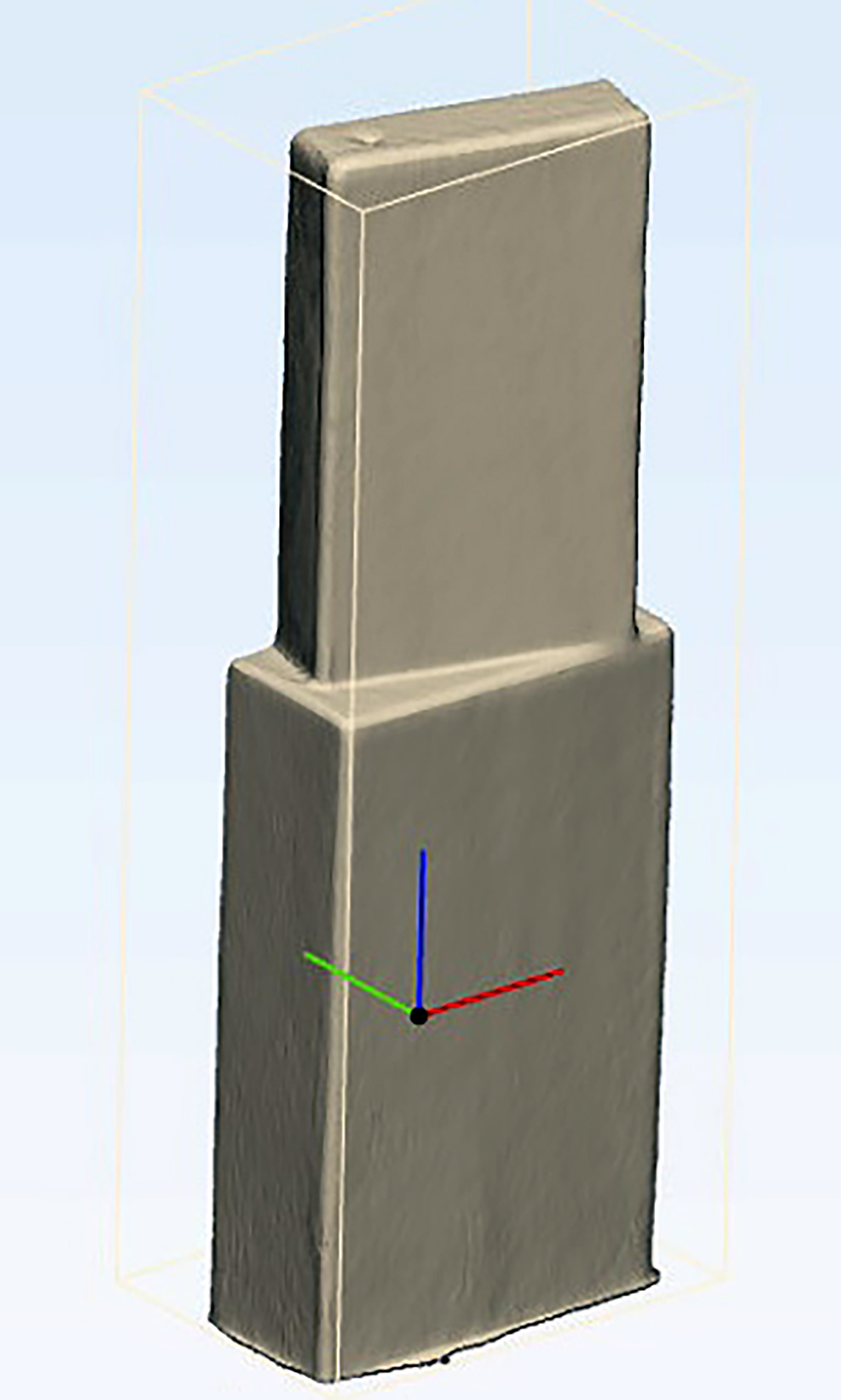

Supplement: S1 Fig — (TIF) [file pone.0229656.s001.tif]
